# Celts under the Knife: Surgical Fortitude, Racial Theory and the British Army, 1800-1914

**DOI:** 10.1080/14780038.2020.1734147

**Published:** 2020-04-14

**Authors:** James Kennaway

**Affiliations:** School of Humanities, University of Roehampton, London, UK

**Keywords:** Surgery, Celts, race, Anglo-Saxons

## Abstract

The nineteenth century saw an extensive cult of the heroic fortitude of the British soldier in surgery. Tales of men laughing through unanaesthetised operations were endlessly repeated in newspapers, military memoirs, surgical literature and boy’s own stories. However, the identity of the “British soldier” was by no means obvious, since the British state itself was multi-ethnic. This paper considers thinking on the ‘natural’ martial qualities of ‘Celtic’ Scottish and Irish soldiers and how they were reflected in discussions of what behaviour could be expected in surgery, considering the impact of cultural explanations, racial theory and implicit political agendas.

The surgeon William Munro, a Highlander by origin, saw service in many of the most iconic moments in the military history of the British Empire under Queen Victoria. He was Assistant Surgeon with the 91st Highlanders in the ‘Kaffir War’ of 1846–7, and then Surgeon to the Sutherland Highlanders when they formed the ‘Thin Red Line’ at Balaklava, and took part in the relief of Lucknow during the so-called Indian Mutiny. After serving as Surgeon-General, he published a memoir in which he described what he perceived as the explicitly British character of the fortitude shown by troops under the care of surgeons during the Crimean War:

And yet the British soldier, in all this misery, never uttered one word of complaint, and was an object to be looked at with admiration and respect … his bearing proud, nay, even majestic, and he looked the picture of a man braving trial and adversity; and from his sunken eye there shone a light which told that his courage still beat high, and that sustained by the courage of his race he would endure “without a murmur” further suffering still, and conquer in the end.^1^

This view of the apparently superhuman fortitude of British servicemen undergoing battlefield surgery as a matter of racial character was very common in books on surgery, military biographies and boy’s own adventures stories throughout the nineteenth century. While the standard narrative of military surgery in the period has tended to focus on the story of the progress of anaesthetics and Humanitarianism in the shape of Florence Nightingale and the Red Cross, the persistence and development of an understanding of the suffering involved as a form of sublime sacrifice was at least as important.^2^ Growing interest in the fate and character of the common soldier led not only to greater attention to his welfare but also to a glorification of his experience of the agonies of amputation and the like, often framed in terms of nation, race and imperialism. Tales of surgical fortitude became a fixed part of a culture of Romantic militarism,^3^ and European colonial empires’ right to rule was regularly justified on the basis of a complex and shifting racial hierarchy of the ability not to inflict but to endure pain, with the men who can suffer it uncomplainingly and exercise political authority at the top and servile imperial subjects, who either experience ‘actual insensibility to pain’ or buckle in the face of agony, at the bottom.^4^

In this context, a central question is what was meant by ‘British soldier,’ since Britishness was by no means a straightforward ethnic designation.^5^ Although the common ‘whiteness’ of British troops appeared to outweigh other considerations in colonial situations, closer to home some observers focused more on the distinctions within the British Isles (itself a controversial term). Since there was no British state at all until 1707, it is fair to say, in Peter Scott’s words, that ‘Britain is an invented nation not much older than the United States.’^6^ The United Kingdom of Great Britain and Ireland was an even more recent phenomenon, created with the Union with Ireland on the first day of the nineteenth century and lasting only a generation into the twentieth. In that period, there was a concerted attempt to construct a unified British identity, but it was only ever partially successful. The English (and others) generally simply continued to call themselves (and often the Scots, Irish and Welsh, too) English, with the rhetoric of Britishness being most common in certain circles in Scotland and in the ‘white’ settler colonies. Although English gradually overwhelmed other languages, millions of men continued to speak Welsh or Irish or Scots Gaelic. Welsh and Scottish nationalism were fairly quiescent in political terms, but they remained potent cultural ideas, albeit generally within a Unionist ideology. Whether Irishness was compatible with Britishness at all was the chief question in Irish (and often in British) politics throughout the century, disputed violently at times.

It is also important to bear in mind is how ‘Celtic’ the army was. In part, this was a reflection of the relative population sizes of the nations involved, which were very different to today’s ratios. Just before the demographic and human catastrophe of the Irish famine of the 1840s, there were probably over eight million people in Ireland and around 2.6 million in Scotland and one million in Wales, many of whom still spoke a form of Gaelic or Welsh as a first language, whereas England could muster around 13.5 million, meaning that ‘Celts’ (broadly understood) made up almost half of the population of the British state – much more than a ‘fringe.’^7^ The state’s multi-ethnic character was explicitly reflected in the army’s unusual system of local regiments with overtly national Scottish, English, Irish or Welsh personalities and uniforms, meaning that soldiers had other conscious identities within the broader British or imperial context. The proportion of non-English troops varied, but remained high. Examining the statistics in 1859, W. Farr, writing in *The Journal of the United Service Institution*, was clear that it would be a ‘mistake’ to think of the ‘English’ army as ‘simple Anglo-Saxons.’ He noted that the number from England (67,647) was about the same as that born in Scotland (15,300) and Ireland (53,169) put together.^8^ Thus, as recent historiography has emphasised, although Britain is sometimes portrayed as a pioneering ‘nation-state,’ it and its army were polyglot, multi-ethnic entities, even before one considers the Empire beyond the ‘home nations.’^9^

The persistent yet fissiparous nature of Britishness was reflected in the discussion of fortitude in surgery, with parallel discussions both on generic ‘British pluck’ and on the specific racial characters of the ‘Anglo-Saxon’ English and their ‘Celtic’ neighbours. This article considers soldiers of Scottish and Irish origin (the Welsh, with much smaller numbers in the army, were not subject to the same level of debate), looking at the the discourse of surgical fortitude about supposedly heroic loyal Highlanders and the more mixed picture of Irish troops. In particular, it looks at the impact of theories of unchangeable inherited racial characteristics on older debates on the role of climate, diet and culture, showing how racial theory affected surgery and how surgery informed racial theory. While those racial theories have been extensively discussed, their relationship to surgery has not yet been illuminated. As this article will show, in a context of complex and fluid political identities, the ‘science’ of racially determined fortitude offered plenty of scope for the projection of agendas related to visions of Noble Savages, nationalism, Unionism and the affective style of sangfroid imperial masculinity. At the same time, the internal contradictions within these British/imperial/Celtic identities meant that these theories were constantly undermined and challenged, in a discourse of scientific racism that was always contested.

## ‘Made of iron’: the anecdotal tradition of Scottish and Irish ‘Celts’ as martial peoples

There was nothing new in the idea that certain peoples were particularly warlike and able to withstand pain. Since Antiquity writers have argued that particular ethnic groups were especially fitted for military conflict and that others, corrupted by city living and luxury, could not stand discomfort, let alone surgery. However, until the modern era, attitudes towards the martial character of particular peoples generally followed an environmental model of causation. The fundamental argument can be found in the Hippocratic text *On Airs, Waters and Places*, which suggested that, ‘the pusillanimity and cowardice of the inhabitants’ of Asia and their ‘unwarlike’ character were partly due to ‘the nature of the seasons’ and that tribes in Europe from areas that were ‘mountainous, rugged, elevated, and well watered, and where the changes of the seasons are very great’ were likely to be of a ‘warlike disposition … and … apt to have no little of the savage and ferocious in their nature.’^10^

The resilience (or lack of it) among ‘Celts’ (i.e. the Irish and the Scots, or at least the Highlanders, and sometimes also the French – the term was always a moving target) was a recurring theme in discussions of bravery in surgery. In line with the Hippocratic view, such thinking often drew on environmental explanations rather than racial theory, especially, but not only, in the first half of the century. For instance, David Stewart of Garth’s influential 1825 *Sketches of the Character, Manner and Present State of the Highlands* implied that the Highlander’s martial character was inherited from clans as a matter of the ‘social system,’ and not of how he was ‘favoured by nature.’^11^ Such notions of the composure of Celts in surgery often owed much to thinking about the poor Celtic rural economy, sometimes put in terms of Enlightenment stadial theories of progress.^12^ Prejudices about the toughness of ‘backward’ peoples formed a key part of the debate on surgical fortitude throughout the nineteenth century and beyond, even though ‘primitive’ warriors were traditionally believed to show great daring in battle but to lack precisely the level of self-control involved in calmly enduring an operation or in maintaing the iron discipline of modern armies.^13^

Some surgical observers made comparisons between English, Scottish and Irish troops that focused on material conditions. Rutherford Alcock, a surgeon in the British forces in the (largely forgotten) military interventions in Portugal and Spain in the 1830s, blamed economic and lifestyle conditions for the differences he saw. The English, he wrote, were ‘a bad class as to physical capacity; a great number of them were sickly Londoners, or men recruited from Liverpool and Bristol, accustomed to the enervating life of a large city.’ The Scots, far from being the strapping Highlanders of the imagination, were ‘chiefly from the manufacturing towns – Glasgow and its neighbourhood’ and also sickly. It was the Irish that ‘were physically and morally the best adapted for the service,’ because, he wrote, they were used to roughing it, as a result of the relative lack of development in Ireland.^14^

Similarly, James Young Simpson, the Scottish pioneer of chloroform, implied that Celts from agricultural backgrounds were likely to have greater powers of endurance in surgery, addressing the issue primarily in terms of diet, even if elsewhere he did indulge in racial speculation. Asking, ‘Is the Irish peasant, fed almost solely on potatoes, or the Scottish peasant fed almost solely upon oatmeal, as liable to fatal surgical fever after surgical injuries?,’ he admitted that he had no evidence to prove it, but said it was the ‘general impression’ that they seem to ‘stand surgical knocks, and injuries, and operations with a wonderful impunity.’^15^ In contrast, he reported that surgeons told him that ‘draymen and other servants’ in London breweries did badly in operations because of their habit of drinking too much ale.^16^ Likewise, in 1844 Irish surgeon D.B. Bullen wrote an article in *The Lancet* that took a systematic approach to try to demonstrate the resilience of the Irish. It suggested that ‘the physical and moral condition of the local population’ was key in survival rates. In particular, it argued that the poor of Cork ‘bear severe operations extremely well’ because of their ‘strong religious feeling’ which gives them that passive courage which does not fear death,' and also due to ‘the vegetable diet upon which the greater numbers live.’^17^

**Figure 1.**
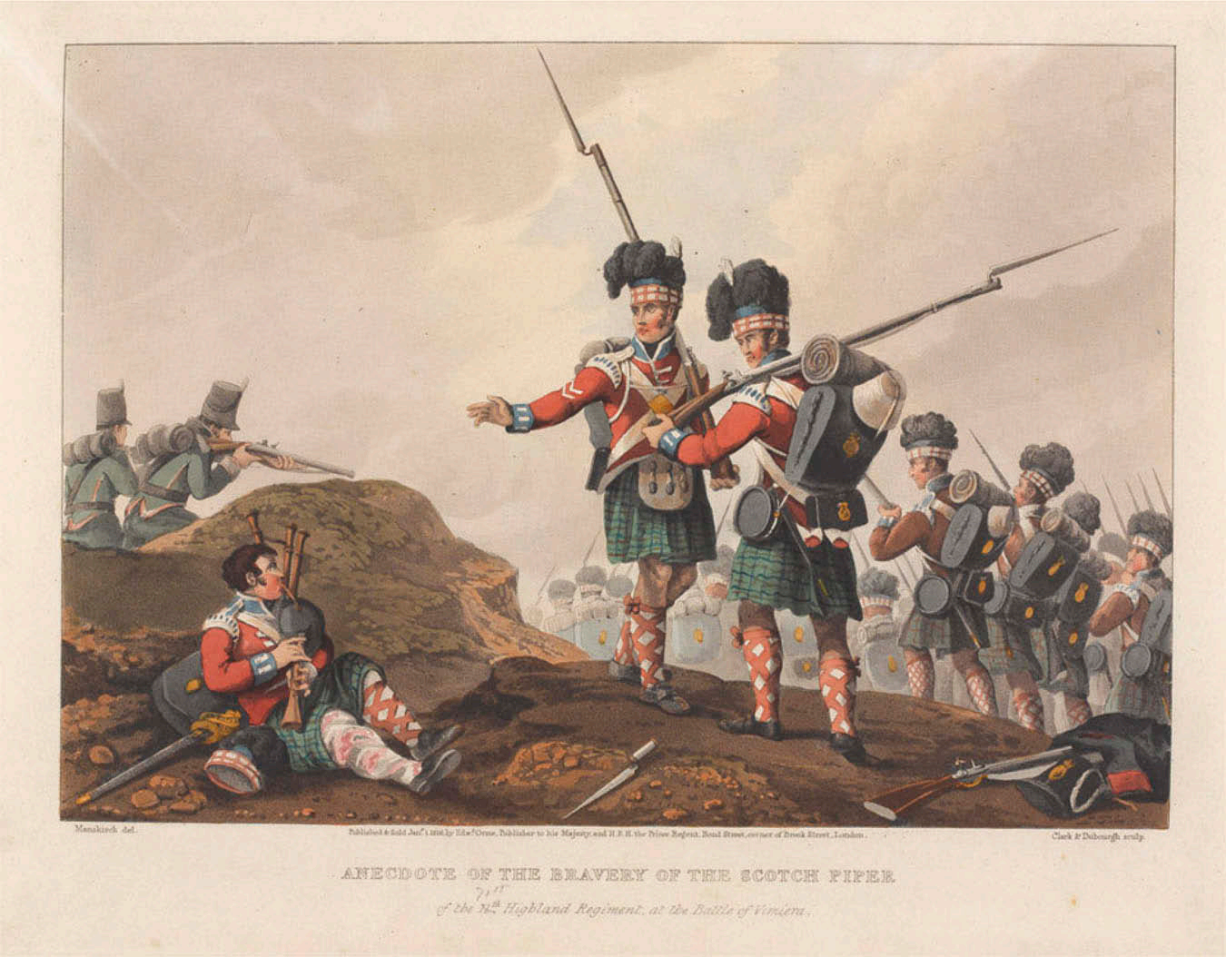
Manskirch – *Anecdote of the bravery of the Scotch piper of the 71st highland regiment, at the Battle of Vimiero* (1819).

As far as Scottish troops, especially Highlanders, are concerned, such theories relating to diet, climate and lifestyle had (and still have) great resonance in explaining their supposed ‘natural’ warlike character and capacity for enduring pain, something that TV, film and romance novels continue to cash in on today.^18^ This mythology of Scots martial prowess is so potent that Hew Strachan has felt it necessary to point out just what a rotten record the Scottish state had in warfare.^19^ Nevertheless, the nineteenth century was marked by a cult of the kilted warrior, sometimes ascribing almost Herculean fortitude to Highland regiments. Indeed, in many ways, with their mountain background, they were a model for the whole ideology of ‘martial races,’ so prominent in debates on the recruitment of Indian troops.^20^ The ability to take pain and losses was generally given a central role in the glorification of Highland troops, as is exemplified in the image of the wounded piper in Figure 1.

The position of Scottish soldiers, the favourite Celts in the British army, was somehwat paradoxical. After being transformed in public perception from Jacobite rebels to loyal British troops, they were subsumed in many ways into an idea of British national character, but at the same time, resplendent with tartan and bagpipes, they achieved worldwide prestige as a syndecdoche for the whole British army.^21^ They were a symbol both of Scottish gallantry and of Unionism and the British Empire – an odd combination of being ultra-British and an exotic Other.^22^ In previous conflicts, notably the Seven Years War, let alone the Jacobite Rebellion, wounded Scots had been of little interest to metropolitan opinion. However, during the Napoleonic Wars the idea of Scottish soldiers as the shock troops of the British state took hold, and military service shifted, in J.E. Cookson’s words, from being a ‘career’ to being a ‘duty informed and inspired … by a quite sophisticated national ideology,’ giving strong impetus to the incorporation of the Scottish soldier in surgery as a British hero.^23^ The Battle of Waterloo was a key point of memory in the following century, and no-one batted an eyelid that the Gordon Highlanders had cried out ‘Scotland for ever!’ or that they had sung *Johnnie Cope* and *Scots wha’ Hae*, patriotic songs about fighting Englishmen.^24^

Tales of Scotsmen scoffing at the agonies of war wounds and medical treatment from were endlessly repeated over the nineteenth century, becoming a key part of this cult of the Highland warrior. For example, Walter Scott, in his 1817 book on the Battle of Waterloo and its aftermath, recorded with Scottish pride the admiration of the locals for Highland soldiers:

“Your countrymen are made of iron,” said a lady in Brussels, and told how she had met wounded Highlander supporting himself by the rails as made his way with difficulty. She said she feared he was very badly hurt and offered help, when he drew himself up, thanked her, and said, “I was born in Lochaber, and I do not care for a wound.”^25^

Similarly, Alexander Mercer, an English artillery officer at Waterloo, recorded a scene in which some troops, either out of cowardice or having been misled ‘by false statements of French success,’ were fleeing the field. When he met a Gordon Highlander who was limping with a bullet in his bandaged knee, Mercer asked for information, telling him what the Belgians had said. ‘“It’s a damned lee. They were fechtin” when I cam’ awa’, an’ they’re fechtin yet.’ He then sat down on a wall and lit his pipe, while the artillery surgeon extracted the bullet.’^26^ Such first-hand testimony often reflects the fact later anecdotes were not merely fantasy, and that ideas about Highlanders’ fortitude, no doubt along with masculine self-esteem and regimental pride, mediated the experience of the men involved.

Regimental histories throughout the period regularly ascribed the apparent natural heroic behaviour of Highlanders in surgery to their ethnicity, but generally in terms of culture and lifestyle rather than inherited unchangeable racial character, turning the real experiences of the men involved into ideologically resonant narratives. Mackay Scobie’s history *An Old Highland Fencible Corps* (1914) looked back with approbation on the way men were ‘taught to endure suffering with a remarkable fortitude and patience,’ including in surgery, where the methods ‘were rough and ready.’ He recorded an instance of Highland endurance from the Napoleonic Wars:

A soldier of the Reays, who had been wounded by a musket-ball which had shattered his arm, was obliged to have the limb amputated by the regimental surgeon. Donald bore the operation with the air of a stoic, and did not utter a word until it was over, when, viewing his severed arm for the last time with a mournful regard, he was heard to express his regret at the loss of a limb which had served him so long and so faithfully!^27^

Nineteenth-century novels also often found tales of almost implausible endurance among Highlanders irresistible, making the Stoical Highlander a stock character. James Grant 1846 novel *The Romance of War, Or The Highlanders in Spain* looked back on the Peninsular campaign, included a scene in which a servant called Jock Pentland is seen dressing a wound in his neck. In reply to the question, ‘Nothing very bad, I hope?,’ Jock replies, ‘“Only a stab in the neck, three inches by one!”’ before refusing to see a surgeon with the resonant phrase, ‘Oh, no! ‘tis a mere scratch.’”^28^

If the Scots were regularly seen as model soldiers, loyal and integrated into the British state and capable of heroism in surgery, Ireland was a much more problematic case.^29^ Although some promoted the idea of Ireland as ‘West Britain,’ many commentators sharply demarcated British identity, supposedly Protestant, rich, industrial, progressive and rational, from a view of Irishness as Other – Catholic, superstitious, rural, poor and potentially disloyal. Irish soldiers’ position was always complicated, caught between their own loyalist traditions and a nationalist critique of them as imperial mercenaries.^30^ The significant role of Irish soldiers in the American Civil War provided an opportunity for a discourse of ‘natural’ Irish surgical fortitude when freed from British power, but other sources stressed their loyalty and endurance in an imperial context.^31^ The ongoing campaigns for and against Irish Home Rule, combined with violent reactions against Fenianism and the mass migration of the Irish to British slums, created a politically charged atmosphere, which made it less than straightforward to depict Irish troops simply as a subcategory within Britishness, even without explicit racial theorising. And in all of this there was the nagging question of who counted as Irish, a question that is still complicated in Northern Ireland and in the Irish Republic’s understandings of the heritage of its own Anglo-Irish former elite.

Essentially environmental explanations of the character of the Irish predominated until the modern period. For instance, in his *Topographica Hibernica*, the twelfth-century Anglo-Norman-Welsh historian Gerald of Wales ascribed their supposed barbarism to their pastoral lifestyle.^32^ Four centuries later, Edmund Spenser’s *A View of the Present State of Ireland* (1596) set out a similar view. He was clear that the Irishman was ‘a very brave souldier,’ with a character suited to withstanding the pain of surgery – ‘hardye, for the most parte greate endurors of colde, labor, hunger, and all hardnes … very present in perills, very greate scorners of death.’^33^ But although he argued for the subjugation of the country and the extirpation of its language and much of its culture, he never suggested that any inherited racial characteristics would be an obstacle to the imposition of English law and discipline.

In contrast to the generally positive nineteenth-century view of Scottish soldiers’ ability to undergo surgery without complaint, there were much more mixed views of how Irishmen could be expected to bear up, even without any racial determinism. Irish soldiers were sometimes simply included under the British banner, with little attention being paid to any specific Hibernian element, but some medical writers expressed very unflattering views of the moral and physical capabilities of the Irish under the knife. In part this was a reflection of dualistic thinking, with the Irish at times being relegated to the role of ‘Other’ that would bring the endurance of British troops out in relief. In his 1840 *Select Discourses on the Function of the Nervous System*, the New York doctor John Augustine Smith, considering the odd phenomenon that some people ‘possessed of courage’ in a ‘remarkable’ degree did not have the ‘passive resolution’ to ‘bear the knife of the surgeon,’ suggested that this applied particularly to the Irish. Despite being ‘the bravest people in Europe,’ he wrote, they were ‘so notorious for their unmanly complaints when on the operation table. The moment the knife touches them, they “cry out murther with a yelping note,” as every attendant of the London Hospitals must have witnessed.’^34^

In contrast, other medical writers defended the honour of Irish soldiers by arguing that they could show great courage in surgery. In the 1840s the surgeon George James Guthrie, who had worked with the army in the Peninsular War and wrote an influential book on gunshot wounds, wrote of a soldier being ‘an Irishman,’ who ‘*therefore* could not flinch, but stood as firm as a rock, whilst the knife, which was not too sharp, grated harshly as it went through the tendinous expansion of his great glutaeus muscle.’^35^ Later conflicts produced many similar stories. The leading English surgeon Frederick Treves wrote a Boer War memoir that includes an account of an Irishman called Kelly, ‘as plucky a soldier as the plucky soil of Ireland has ever produced.’ Having had his right arm ‘smashed,’ it was necessary to amputate the whole upper limb,which he accepted ‘with infinite courage and with irrepressible good humour.’^36^

**Figure 2.**
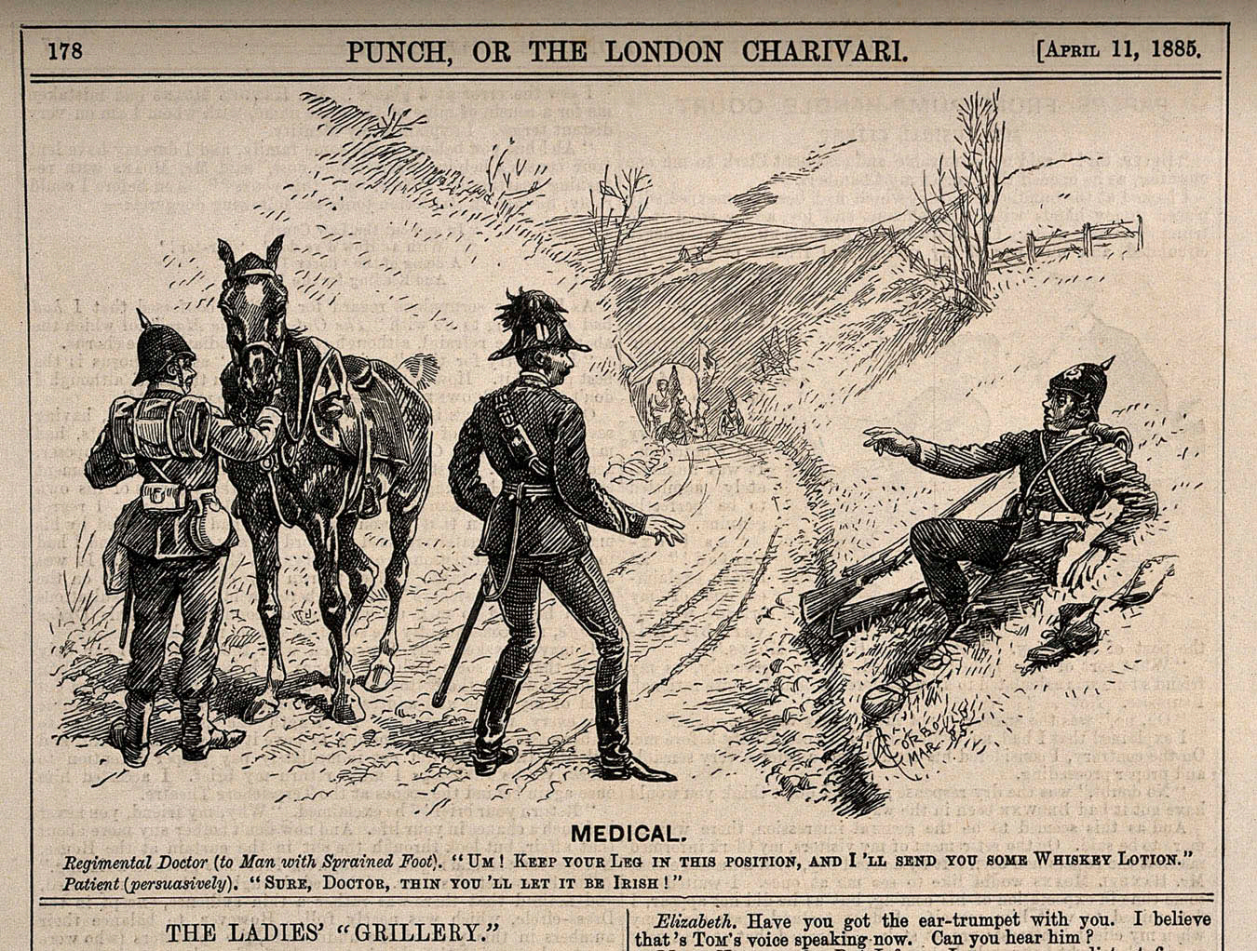
*Punch –* ‘Medical’ (1885).

**Figure 3.**
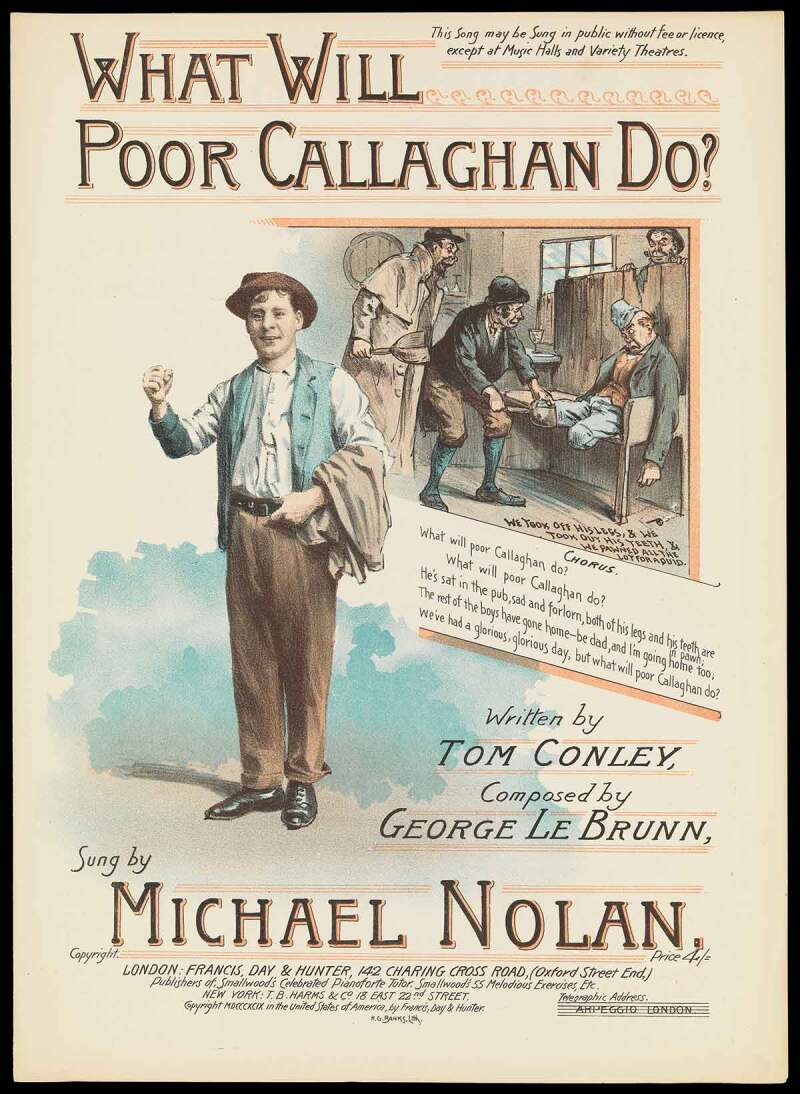
Tom Conley and George le Brun – *W@hat Would Poor Callaghan Do?*

Humour was often a theme in descriptions of Irish private soldiers’ sufferings, often combining genuine admiration with patronising depictions of their accents and supposedly childlike or foolish behaviour, particularly in the second half of the nineteenth century. An image from an 1885 edition of *Punch* shows a wounded Irish soldier demanding Irish whiskey of the regimental doctor who offered him a whiskey lotion (see Figure 2). And the music hall song, ‘What Will Poor Callaghan Do?’ was about four Irishmen who get an Irish ex-soldier (a veteran of a Highland regiment) drunk and pawn his wooden leg (see Figure 3). Scenes from military and surgical memoirs sometimes reflected a similar attitude, often verging on parody. For instance, Nathaniel Steevens’ *The Crimean Campaign with the Connaught Rangers* records an Irishman called McCann who had a leg amputated remarking to a comrade, ‘If I lose my leg I will get 1 s. day pension,’ not being then aware that his leg was off.^37^

## Scientific racial theory and the martial races of Britain in surgery

By the mid-nineteenth century this anecdotal style of discourse on Celtic troops’ resilience in surgery described above was joined by a more ‘scientific’ view that saw the putative differences between categories of soldiers as the result of unalterable inherited characteristics.^38^ As several historians including Peter Mandler have recently emphasised, these scientific racial theories were never unchallenged, and older traditions of a focus on cultural rather than biological difference and on the ‘ladder of civilisation’ remained influential even after the hardening of attitudes in the wake of the Indian Mutiny and Jamaican Rebellion and the rise of Darwinism.^39^ Nevertheless, the term ‘race’ did often take took on a harsher and more biological sense, drawing on work by physical anthropologists and anatomists such as Pieter Camper, Johann Friedrich Blumenbach and John Cowles Prichard.^40^ Moving towards a more fixed conception of Celtic identity based on heredity and racial determinism both influenced surgical thinking and gave surgeons a role in the creation of racial knowledge. It also had clear connotations in politics, since ascribing different levels of physical sensitivity and moral strength in relation to pain to environmental causes implied a view that left open a liberal imperialist option of future self-government. Unchanging racial hierarchies, in contrast, offered a more direct justification of indefinite domination – a factor that had more drastic implications in parts of the Empire with harder racial boundaries.

While there had been plenty of allusions to the common ancestry of the Scots and Irish since the Middle Ages, the word Celt only came to be used as an umbrella term for the non-English peoples of the British Isles in the early eighteenth century. As many observers have argued, the term ascribes an illusory unity to peoples spread over centuries who had no idea of the commonalities of language perceived by scholars.^41^ Nevertheless, the rise of racial theory, drawing on the linguistic investigations of the eighteenth and nineteenth centuries, led to the scientific study of the ethnic traits shared by a supposedly common people.^42^ Those theories remained highly speculative and contradictory, since, in the absence (and, indeed, impossibility) of clear demarcations, all of the categories involved were unstable.

A key figure in debates on racial theory was the Edinburgh anatomist Robert Knox. He studied medicine in Edinburgh and Paris, working as a surgeon at Waterloo and with the 72nd Highlanders in Africa.^43^ He had a particular interest in the ethnic make-up of the United Kingdom, with the ‘Saxon’ English and Lowland Scots at the top and the Celtic Irish and Highland Scots as an inferior and dangerous alien breed. As far as martial qualities are concerned, Knox argued that while ‘the Saxon’ has natural ‘strength and obstinacy,’ he despises the servile status of a soldier, but the ‘Celt makes the best of soldiers’ because he is ‘destitute of all self-esteem.’^44^ ‘The pugnacious irritability of the Irishman, the Welshman and the Highlander, has always been proverbial,’ he argued.^45^ While Douglas Lorimer is right to note that Knox was an intellectual ‘outsider’ in his own generation, many observers in the second half of the century were more open to such thinking. As Knox’s biographer Henry Lonsdale put it in 1870, ‘Those who felt disposed to laugh in 1846 at Knox’s theories of Race, were surprised at the historical endorsement they obtained in 1860.’^46^

Much of the racial debate on the resilience to be expected of Celts posited English soldiers as the default, with Celts as a variation, sometimes heroic, but also sometimes as Noble Savages or as crude outsiders. Although some scholars still play down the influence of English racial thinking, there was an intensive if controversial debate about the racial character of the English.^47^ The nineteenth century was the golden age of ‘Anglo-Saxonism’ as an ideology – and not just in England, even if that discourse also remained contested and confused, with books such as Luke Owen Pike’s *The English and their Origin* (1866) suggesting the English were ‘more Celtic than Teutonic.’^48^ Many observers in previous generations had found it easy to admit that the English were a mongrel breed, but by the Victorian era this idea was being challenged by a sense that the English were in essence pure Teutons. Earlier thinking on England’s Germanic origins had often focused on the supposed egalitarianism of the Saxons before the ‘Norman Yoke,’ but ‘Saxonism’ was increasingly associated with racial theories of imperial domination, as seemingly unstoppable Protestant Anglophones looked for a scientific rationale for their expansion.

Discussions of the powers of endurance among the ‘Saxon’ English were common among racial theorists of the period in way that was relevant for surgery. American racial theory was ahead of the game. In his influential *Crania Americana* (1839), the Philadelphia racial theorist Samuel George Morton argued that ‘the moral character’ of the Germanic peoples was marked ‘by decided personal courage, great endurance of fatigue, firmness and perseverance.’^49^ The English physicians and ethnologists Joseph Bernard Davis and John Thurnam expressed similar assumptions in their later 1865 *Crania Britannica*, suggesting that since the Norfolk Militia were characterised by a high level of Viking blood, they could be expected to show ‘a determined courage which knows no fear nor when beaten, and great perseverance and power of endurance.’ In a revealing note, they also implied that this capacity for enduring pain was linked to their supposedly racially determined political independence: ‘They are patient, not easily excited, but when stirred up very difficult to be put down, are to be controlled by kindness, but of too independent a spirit to be harshly dominated over.’^50^

The question of the powers of endurance in surgery of Celts as a biological race presents a mixed and indeed contradictory picture. In an 1858 article surveying the condition of the army after the Indian Sepoy Rebellion, Colonel W.H. Sykes wrote in racial terms that the Celts of the British Army possessed not only ‘great bodily strength, bravery, coolly and steadily maintained’ but also ‘great bodily and mental powers of endurance of adversity and of pain.’ This was in contrast to their supposed fellow Celts in France, who, while demonstrating ‘greater intelligence and quickness, and often as much courage,’ cannot ‘bear adversity so long or so patiently.’^51^ In contrast, the English physiologist Thomas Laycock, the Yorkshire-born Professor of Medicine in Edinburgh from 1855 (a man who refused anaesthetic when he had a leg amputated), was less positive. In the 1860s, using his own experience treating patients as the epistemological basis of understanding their racial character, he contrasted the English ‘Saxons,’ who he said were ‘vigorous, healthy … not excitable or hasty in action, but … characterised rather by stolidity of temper,’ with Celts, who showed ‘pathological tendencies,’ that were ‘emotional or feminine’ (rarely a positive word in race hierarchies).^52^

Clinically you will find the Celt more difficult to examine than the Saxon, on account of the activity of his imagination …. There is greater susceptibility to all impressions; greater predisposition to excitation by all nervous stimuli … In general we find the Irish or Highland patient is less hopeful and more apprehensive; and from his keener susceptibilities is less tolerant of painful diseases, is more demonstrative of his feelings, and requires more manifestation of sympathy and of interest in his case.^53^

Laycock set out his ideas about the physiological and moral weakness of Celts facing pain in the context of his broader poor opinion of them as a race, blaming what he saw as their religious errors and political folly on natural racial flaws.^54^

Who exactly counted as a Celt, though? Were Scots Celts at all? Or only Highlanders? Again, the contradictions involved made the creation of a coherent theory of Celtic fortitude complicated. Modern Scottish nationalism, and the historiographical topos of the ‘Celtic Fringe’ has sometimes led to an exaggerated sense of the similarity of Scotland, Ireland and Wales. An explicitly Lowland, Unionist, Presbyterian and often anti–Irish Scottish identity was mainstream, fostering a form of Scottish patriotism very different from that which has developed in recent decades.^55^ As Colin Kidd and others have noted, many nineteenth-century Lowland Scots were positively aggressive in asserting their Germanic and even ‘English’ character and in distinguishing themselves from the impoverished and marginalised Gaelic-speaking population.^56^ The influence of scientific racism thus tended to lead to a focus on the Lowland/Highland distinction rather than the Anglo-Scottish border as the real division when it came to discussing powers of surgical endurance, even as the identity of Lowland soldier came to be subsumed in that of the mighty Highlander in the popular imagination.

For example, the surgeon William Fergusson suggested that, ‘the Lowland Scot, is of the same Saxon breed as his southern neighbour.’ Conceding while he was ‘inferior’ to the Englishman in physique and appearance, he argued that a rougher life (i.e. environmental causes) made him hardier – ‘uniformly patient, obedient, and brave, never murmuring under any suffering.’^57^ However, he saw a sharp distinction between Lowland and Highland Scots characterised by inherited characteristics. ‘The Highlander (the Celt) comes from a different stock, and it is difficult to conceive two races of men, separated often by the narrowest, or even imaginary boundaries, more distinct than the mountaineer and his lowland neighbour.’ The Highlander, he wrote, has a peculiar aptitude for war, being capable of ‘the most exalted heroism,’ inspired by the ‘horrible’ sound of bagpipes, but being otherwise as ‘slothful and indolent as the North American Indian.’^58^

A further flaw in the popular notion that Stoical Scottish soldiers were in essence Celtic and Highlanders was the sharp decline in the numbers of actual Highlanders in the army. The period in which recruitment in the Highlands peaked was comparatively short, from the 1750s to the 1820s. It was the Napoleonic Wars that set the seal on the reputation of Highland troops, with somewhere between 37,000 and 48,000 Highlanders recruited. Thereafter, improved prospects in booming industrial cities at home and increased access to land in the British Empire and United States meant that fewer of the men of the Highlands chose a military career. The 1881 Army reforms, which made the visual aspect of Lowland regiment align with those in the Highlands, bringing in the kilt, bagpipe and tartan, obscured but did not end the decline in recruitment in the Highlands.^59^ Nevertheless, the advent of thoroughgoing racial theory in the second half of the century played a significant role in the trajectory of the reputation of the Highland soldier from the supposed undisciplined ‘bare-arsed banditti’ of the 1745 Rebellion to the emblem of the British Army.^60^ Assuming that their apparent toughness in battle and in surgery was a matter of inherited racial characteristics reinforced the idea that Highlanders were natural soldiers for the Empire – something aided by the smoke and mirrors of tartanry used to suggest a Highland character to a group of men increasingly drawn from other areas.

However, beyond the fact that such racial thinking is both scientifically discredited and politically bankrupt, it is also the case that the ‘natural’ martial valour often ascribed to Highland troops was only one side of a race theory that also often blamed the poverty and misery of the Highlands on those same racial characteristics. An 1859 article in *The Morning Post* on the Scottish Poor Law Board was fairly typical in arguing that ‘the influence of race’ explained the fact that Highlanders were ‘gallant in war,’ but that their lack of prosperity was due to the fact they were ‘a toil-hating race.’^61^ This at a time of considerable distress and drastic depopulation in the Highlands. As Alexander Mackenzie pointed out in his 1883 *The History of the Highland Clearances*, many of the ‘brave fellows’ who came back ‘wounded and war-scarred,’ whether in combat or surgery, found ‘that their hearths and homes were desecrated and destroyed.’^62^

As far as Ireland is concerned, the decades following the 1798 Rebellion saw attempts to explain the character of Ireland in terms of uneradicable racial nature. Writers such as Goldwin Smith, Tuthill Massy and Daniel Mackintosh drew on historical linguistics, physical anthropology and anatomy to set out their views of the natural characteristics of the Irish.^63^ However, as scholars such as Perry Curtis have shown, depictions of the Irish as almost a separate species, an almost apelike creature, ‘prognathous-jawed – the negro type,’ emerged only in the mid-nineteenth century.^64^ Such thinking also provided a more systematic view of the racial basis of Irish behaviour in surgery. Some observers who were wedded to racial theory were happy to acknowledge the composure of Irish troops in surgery, interpreting their own experience in the light of race science. William Fergusson may have feared that ‘Paddy’ was ‘ungovernable’ and ‘childlike,’ but he praised his ‘high renown’ and wrote that he was characterised by ‘hardihood, courage, and endurance.’^65^ However, another group of medical observers took the opposite view, seeing Irish soldiers as too emotional and temperamental to show heroic endurance in surgery. Joseph Bernard Davis and John Thurnam’s *Crania Britannica* was not flattering about the ‘primeval’ Irish of the Gaelic-speaking west of the island, noting their natural ‘treachery.’ They argued that the inborn inadequacies of the Irish led to ‘fatalism,’ which made them express ‘unreasonable alarm’ and a ‘dread of pain’ in sickness – the basis of moral and physical weakness under the knife.^66^

As a number of scholars have shown, the advent of effective anaesthetics led to an extensive debate on the meaning and possible value of pain, most famously in relation to women in labour. It also produced a wide discussion on how different races would respond to anaesthesia and which could endure pain, including with regard to Celts.^67^ The Dublin surgeon Rawdon Macnamara included a long discussion of nationality and chloroform in his *Neligan’s Medicines* (1867), which explained the apparent differences in fortitude under the knife in terms of natural racial character. He contrasted ‘Saxon Englishman, pur sang,’ with ‘the more vivacious, mercurial, and nervously excitable temperaments of the French and Irish specimens of the great Celtic brotherhood.’^68^ Despite being himself an Irishman, Macnamara provided a harsh judgement on ‘the Hibernian Celt’ in particular. The Irish soldier, he argued, was responsible for

the most marked exhibition of … chloroformic excitement, and its wildest demonstrations. Inhalation was stoutly resisted, and when partially effected, soon gave evidence of its exciting and intoxicating effects by furious struggles, curses ‘both loud and deep,’ anger in one case, risible excitement in another, and ﬁnally a voluble outpouring of the native Irish … . The amount of time consumed in such cases before the patient was reduced to a condition of anaesthetic quiet was in many instances very great, and became the source of inﬁnite trouble.^69^

Despite speculation about the racial unity of the Celts, the different political and cultural contexts in Ireland and Scotland ensured that contradictory evidence was put forward. Macnamara considered that the ‘Scotch Celt’ exhibited only to ‘a partial degree the excitement so marked in his Irish brother.’^70^ Likewise, Thomas Watson’s *Lecture on the Principles and Practice of Physic* (1857) noted a contrast in the ‘different degrees of patience’ shown by supposed fellow Celts in Ireland and Scotland. ‘The Irishman,’ he suggested, ‘either feels more acutely, or gives more free vent to his feelings in cries and exclamations,’ but ‘the Scotchman’ could be expected to preserve ‘a resolute silence.’^71^ However, in a measure of the confusion involved in such discussions, William Fergusson, a Scot, wrote that, ‘the most querulous under wounds and sickness have been the Scotch Highlanders. The Irish may be more noisy, but then it is with less plaint.’^72^ A third group was sceptical of racial determinism altogether. For instance, the Scottish Liberal MP and journalist John Mackinnon Robertson’s *The Saxon and the Celt* (1893) attacked the ‘fallacy of race prejudice’ in this context.^73^ Such voices, dubious not only of the anti–Irish or anti-Celtic agenda but of the whole discourse of scientific racism, continued to be raised throughout the period, reflecting the fact that these racial categories often seemed in danger of collapsing under the weight of fundamentally inconsistent stereotypes.

In the heated political context of Ireland, the implications of the racial theorising that included arguments for the natural resilience or weakness of Irish troops in surgery were even more hostile than in the Highlands. Robert Knox, that pioneer of racial theory, expressed ominous attitudes in the 1840 s towards the Irish on race grounds, suggesting that the ‘Saxon blood’ in Ireland (‘the Orange party’), ‘should be allowed and encouraged to expel the Celtic race and send them to America.’^74^ Knox foresaw a future like that predicted for the American Indian – that the ‘Celtic’ Irish should die out, defeated in the struggle for life. An anonymous article in *The American Literary Magazine* in 1847 made a similar argument, noting the ‘resemblance’ between native Americans and ‘the Hiberno-Celtic family.’^75^ Like the American Indian, it was suggested, ‘the Irish peasant’ was notable for his passive courage.^76^ In this context, assumptions about Irish soldiers’ powers of endurance in surgery were closely linked to their claims to political self-determination. As was the case with many peoples beyond Europe, implicit arguments for the political subordination of the Irish could be drawn from the suggestion that they were too weak psychologically and physically to endure the agonies of surgery, and if they were assumed to be resilient, that could be put down to their primitive insensitivity.

## Conclusion

It seems, then, that the racial discourse of Celtic composure in surgery had a number of different conceptions of the peoples concerned. Often Scottish Highlander and Irish troops were portrayed simply as a ‘local colour’ variation on a British theme, and their heroics under the knife depicted as part of the superior moral and physical power underpinning whole British imperial project. At other times, so-called Celtic troops were represented either as an alien subaltern breed of warlike supermen or as a childish, weak and mercurial bunch, incapable of the the physiological toughness or psychological grandeur needed for fortitude in surgery or for political self-determination. In line with the half-completed integration of the ‘Celtic Fringe’ into the British state, Highlanders and Irish troops were, in different ways, in an ambivalent position as far as attitudes towards their surgical endurance are concerned, partly mercenaries for a ‘foreign’ power, exploited as volunteers generally escaping from poverty, but also partly beneficiaries, with a position in a racial hierarchy and prestige within the British National Romance.

In their discussions of Celts in memoirs and theoretical works, surgeons drew on a longstanding anecdotal tradition of tough ethnic groups related to environmental conditions. By the middle of the nineteenth century, though, racial theory had clearly penetrated surgical discourse, offering a supposedly scientific basis in heredity for perceived differences. Surgeons also drew on what they thought they had learned from direct personal experience of patients – a source of knowledge that inevitably remained impressionistic, providing an additional source for race theories in surgical observation. Although evidence is hard to come by, it is not perhaps implausible to suggest that this sense that they could distinguish levels of pain between racial groups may on occasion have had an effect on the treatment Celtic troops received – it is certainly the case that recent studies have demonstrated that racial thinking affects the level of pain relief given to twenty-first-century patients.^77^ Racial visions of heroism in surgery were never without its critics, however. This was partly a matter of the continuing influence of traditions of cultural explanation for ethnic differences of the kind set out by scholars such as Mandler, but was also the result of the contradictions between notions of Celtic racial unity and the contrasting political, cultural and social situations in Ireland and in Highland and Lowland Scotland that militated against the creation of a coherent ideology of Celtic racially determined fortitude in surgery.^78^ A single soldier could be Celtic, British, imperial, Unionist, white, feminine, emotional, brave and Stoical, depending on context – and these debates on surgical fortitude starkly revealed those contradictions as much as they reinforced assumptions about Celts. An examination of the debate on the surgical fortitude of Celts thus shows that such ideas were always disputed, and that there were always observers who argued that reactions to the agony of surgery had little to do with race (even if culture can have a measurable impact on reported pain) – that, in the words of Robert Burns, ‘A man’s a man for a’ that.’

## Notes

1. William Munro, *Reminiscences of Military Service with the 93rd Sutherland Highlanders* (London: Hurst and Blackett, 1883), pp. 53–4.

2. See Yuval Noah Harari, *The Ultimate Experience: Battlefield Revelations and the Making of Modern War Culture, 1450–2000* (Basingstoke: Palgrave, 2008).

3. See Nancy Rosenblum, ‘Romantic Militarism’, *Journal of the History of Ideas*, 43.2 (1982), p. 249–68.

4. Ferdinand Werne, *African Wanderings; Or, An Expedition from Sennaar to Taka, Basa and Beni* (London: Longman, Brown, Green, and Longmans, 1852), p. 137. See also Heather Streets, *Martial Races: The Military, Race and Masculinity in British Imperial Culture* (Manchester: Manchester University Press, 2004).

5. See Evan Gottlieb, *Feeling British: Sympathy and National Identity in Scottish and English Writing, 1707–1832* (Lewisburg: Bucknell University Press).

6. Peter Scott, *Knowledge and Nation* (Edinburgh: Edinburgh University Press, 1990), 168.

7. In 2019, in contrast, Ireland, Scotland and Wales together have about 15 million people, about a quarter of the 55.6 million in England.

8. The Navy was much less ‘Celtic’ in character, having 23,401 English, 9,466 Scots and only 3,036 Irishmen. W. Farr, ‘The Application of Statistics to Naval and Military Matters’, *The Journal of the United Service Institution*, 3 (1859), pp. 209–24, here 211. See also Mary A. Conley (ed.), *From Jack Tar to Union Jack: Representing Naval Manhood in the British Empire, 1870–**1918*; and Joanne Begiato, ‘Tears and the Manly Sailor in England, c. 1760–1860ʹ, *Journal for Maritime Research*, 17.2 (2015), pp. 117–33.

9. See John M. MacKenzie, ‘Irish, Scottish, Welsh and English Worlds? A Four‐Nation Approach to the History of the British Empire’, *History Compass* 6.5 (2008), pp. 1244–63.

10. Hippocrates, *On Airs, Waters, Places* (London: Wyman and Sons, 1881), pp. 97–101.

11. David Stewart of Garth, *Sketches of the Character, Manner and Present State of the Highlands* (Edinburgh: Archibald Constable, 1825), pp. 1.242–3. Tom Devine has strongly argued that the Highland soldier of that period was not a sign of continuity with the clan past, but rather the result of a proto-capitalist system of patronage. See Tom Devine, *The Scottish Nation* (Harmondsworth: Penguin, 2012), p. 185.

12. See Keith Thomas, *In Pursuit of Civility* (New Haven: Yale University Press, 2018), p. 184.

13. See John Mackinnon Robertson, *The Saxon and the Celt: A Study in Sociology* (London: University Press, 1893), pp. 128, 270–1.

14. Rutherford Alcock, *Notes on the Medical History and Statistics of the British Legion in Spain* (London: John Churchill, 1838), p. 4.

15. James Young Simpson, *Clinical Lectures on the Diseases of Women* (Philadelphia: Blanchard and Lee, 1863), p. 174.

16. Ibid.

17. D.B. Bullen, ‘On the Results and Treatments of the Cases of Amputation Performed at the Cork North Infirmary’, *The Lancet*, 25.1 (7 September 1844), pp. 720–5, here 720–1.

18. See Peter Womack, *Improvement and Romance: Constructing the Myth of the Highlands* (London, 1989); Kenneth McNeil, *Scotland, Britain, Empire: Writing the Highlands, 1760–1860* (Columbus, 2007); Robert Clyde, *From Rebel to Hero: The Image of the Highlander, 1745–1830* (East Linton, 1995); Trevor Royle, ‘From David Stewart to Andy Stewart: The Invention and Re-Invention of the Scottish Soldier’, in Ian Brown (ed), *From Tartan to Tartanry: Scottish Culture, History and Myth* (Edinburgh: Edinburgh University Press, 2010), pp. 48–62; Edward M. Spiers, *The Scottish Soldier and Empire 1854–1902* (Edinburgh: Edinburgh University Press, 2006).

19. Hew Strachan, ‘Scotland’s Military Identity’, *Scottish Historical Review*, 85 (2006), pp. 315–32.

20. Streets, *Martial Races*, pp. 55–62.

21. John Mackenzie, ‘Empire and National Identities: The Case of Scotland’, *Transactions of the Royal Historical Society*, 8 (1988), pp. 215–231, here 217.

22. Tom Devine, ‘Soldiers of Empire, 1750–1914’, in John M. Mackenzie and T.M. Devine (eds), *Scotland the British Empire* (Oxford: Oxford University Press, 2011), pp. 176–95; Tom Devine, *Scottish Clearances* (London: Allen Lane, 2018), pp. 253–66.

23. J.E. Cookson, *The Armed Nation, 1793–1815* (Oxford: Clarendon, 1997), p. 16.

24. John Dickson, ‘The Battle of Waterloo as Seen by an Ordinary British Cavalryman’, in Mackenzie Macbride (ed), *With Napoleon at Waterloo and other Unpublished Documents of the Waterloo and Peninsular Campaigns* (Philadelphia: JB Lippincott, 1911), pp. 138–48; See also Lt Colonel Wyndham, ‘Reminiscences of the Battle of Waterloo’, 13 August 1839, p. 3. (Royal Scots Museum Archive G163).

25. Walter Scott, ‘Paul’s Letters to his Kinfolk’, in *The Miscellaneous Prose Work of Sir Walter Scott*, 3 vols (Edinburgh: Robert Cadell, 1867), pp. 1.441–524, here 1.482.

26. Cited in C. Greenhill Gardyne, *The Life of a Regiment: The History of the Gordon Highlanders* (Edinburgh: David Douglas, 1901), p. 429.

27. I.H. Mackay Scobie, *An Old Highland Fencible Corps* (Edinburgh: William Blackwood, 1914), p. 200.

28. James Grant, *The Romance of War, Or The Highlanders in Spain* 4 vols. (London: Henry Colburn, 1846), pp. 2.166–7.

29. Keith Jeffrey, ‘The Irish Military Tradition and the British Empire’, in Keith Jeffrey (ed), *An Irish Empire? Aspects of Ireland and the British Empire* (Manchester: Manchester University Press, 1996), pp. 94–122.

30. See Terence Denman, ‘Ethnic Soldiers Pure and Simple’? The Irish in the Late Victorian British Army’, *War in History*, 3.3 (1996), pp. 253–73. Catriona Kennedy, ‘“True Brittons and Real Irish”: Irish Catholics in the British Army during the Napoleonic Wars,’ in Catriona Kennedy and Matthew McCormack (eds), *Soldiering in Britain and Ireland, 1750–1850* (Basingstoke: Palgrave, 2013), pp. 37–56; Peter Karsten, ‘Irish Soldiers in the British Army, 1792–1922: Suborned or Subordinate?’ *Journal of Social History*, 17.1 (1983), pp. 31–64, here 54. Thomas E. Jordan, ‘Queen Victoria’s Irish Soldiers: Quality of Life and Social Origins of the Thin “Green” Line’, *Social Indictors Research*, 57.1 (2002), pp. 73–88.

31. See, for instance, David Power Conyngham, *The Irish Brigade and its Campaigns* (Glasgow: Cameron and Ferguson, 1868), pp. 109, 123.

32. Gerald of Wales, *The History and Topography of Ireland* (Harmondsworth: Penguin, 1982), p. 101.

33. Edmund Spencer, *A View of the Present State of Ireland* (Oxford: Clarendon, 1970), p. 72.

34. John Augustine Smith, *Select Discourses on the Function of the Nervous System, in Opposition to Phrenology, Materialism and Atheism* (New York: D. Appleton, 1840), pp. 14–15.

35. George James Guthrie, ‘A Course of Lectures on Some of the More Important Points in Surgery’, *The Medical Times* 19 (October 1848–June 1849), pp. 85–88, here 85. Italics mine.

36. Frederick Treves, *The Tale of a Field Hospital* (London: Cassell and Company, 1912).

37. Nathaniel Steevens, *The Crimean Campaign with the Connaught Rangers* (London: Griffith and Farran, 1878), p. fn266.

38. See Suman Seth, *Difference and Disease: Medicine, Race and the Eighteenth-Century British Empire* (Cambridge: Cambridge University Press, 2018).

39. See Douglas Lorimer, *Science, Race Relations and Resistance: Britain 1870–1914* (Manchester: Manchester University Press, 2013), Peter Mandler, ‘Race and “Nation” in mid-Victorian Thought’, in Stefan Collini, Richard Whatmore and Brian Young (eds), *History, Religion and Culture: British Intellectual History 1750–1950* (Cambridge: Cambridge University Press, 2000), pp. 224–244, Catherine Hall, ‘The Nation within and Without’, in Catherine Hall, Keith McClelland and Jane Rendall (eds), *Defining the Victorian Nation: Class, Race, Gender and the British Reform Act of 1867* (CUP, 2000), pp. 179–233.

40. See Nancy Stepan, *The Idea of Race in Science: Great Britain 1800–1960*, London (Macmillan, 1982); George W. Stocking, *Victorian Anthropology* (New York: Macmillan, 1987).

41. See Murray Pittock, *Celtic Identity and the British Image* (Manchester: Manchester Unversity Press, 1999), Barry Cunliffe, *The Ancient Celts* (Harmondsworth: Penguin, 1999).

42. See, for example, John Pinkerton, *Dissertation on the Origin and Progress of the Scythians or Goths, being an Introduction to the Ancient and Modern History of Europe* (London: John Nichols, 1787), James Cowles Prichard, *Eastern Origin of the Celtic Nations* (Oxford: S. Collingwood, 1831).

43. Henry Lonsdale, *A Sketch of the Life and Writings of Robert Knox, the Anatomist* (London: Macmillan and Co., 1870), p. 288–9.

44. Robert Knox, *The Races of Men* (London: Henry Renshaw, 1863), p. 472.

45. George Ellis, *Irish Ethnology* (Dublin: Hodges and Smith, 1852), p. 29. The ‘natural’ martial character of the ‘Celtic’ French was proverbial until their catastrophic defeat to the Prussians in 1870, from which point the same was said of the Germans.

46. Douglas Lorimer, *Science, Race Relations and Resistance: Britain 1870–1914* (Manchester: Manchester University Press, 2013), pp. 60, 64; Henry Lonsdale, *A Sketch of the Life and Writings of Robert Knox, the Anatomist* (London: Macmillan and Co., 1870), p. 317.

47. See Peter Mandler, *The English National Character: The History of an Idea from Edmund Burke to Tony Blair* (New Haven: Yale University Press, 2006), p. 72.

48. Luke Owen Pike, *The English and their Origin* (London: Longman, Green, and Co., 1866), p. 64. See Reginald Horsman, ‘Origins of Anglo-Saxonism in Great Britain before 1850’, *Journal of the History of Ideas*, 37.3 (1976), pp. 387–410. L.P. Curtis, *Anglo-Saxons and Celts* (Bridgeport: University of Bridgeport, 1968).

49. Samuel George Morton, *Crania Americana* (Philadelphia: John Pennington,1839), p. 13.

50. Joseph Bernard Davis and John Thurnam, *Crania Britannica* (London: For Subscribers, 1865), p. 219.

51. Colonel Sykes, ‘Our Army in India’, *The North British Review*, 34 (1858), pp. 211–43, here 211.

52. Thomas Laycock, ‘Clinical Lecture on the Physiognomical Diagnosis of Diseases’, *The Medical Times and Gazette*, 1 (1864), pp. 101–3. See also Rosalind Carr, ‘The Gentleman and the Soldier: Patriotic Masculinities in Eighteenth-Century Scotland’, *Journal of Scottish Historical Studies*, 28.2 (2008), pp. 102–21.

53. Thomas Laycock, ‘Clinical Lecture on the Physiognomical Diagnosis of Diseases’, *The Medical Times and Gazette*, 1 (1864), pp. 101–3.

54. See Thomas Laycock, ‘A Chapter on Some Organic Laws of Memory’, *The Journal of Mental Science*, 94 (1875), pp. 155–87, here 187.

55. For the historiographical debate on Scottish national identity, see Hugh Trevor-Roper’s polemical and controversial ‘The Invention of Tradition: The Highland Tradition of Scotland’, in Eric Hobsbawm and Terence Ranger (eds), *The Invention of Tradition* (Cambridge: Cambridge University Press, 1983), pp. 15–42; Peter Mandler, ‘What is “National Identity”? Definitions and Applications in Modern British Historiography’, *Modern Intellectual History*, 3 (2006), pp. 271–97; D. Forsyth, ‘Empire and Union: Imperial and National Identity in nineteenth-century Scotland’, *Scottish Geographical Magazine*, 113.1 (1997), pp. 6–12; Neil Davidson, *The Origins of Scottish Nationhood* (London, Pluto Press, 2000).

56. Colin Kidd, ‘Race, Empire and the Limits of nineteenth-century Scottish Nationhood’, *The Historical Journal*, 46.4 (2003), pp. 873–92; Colin Kidd, ‘Teutonist Ethnology and Scottish nationalist inhibition, 1780–1880’, *Scottish Historical Review*, 124 (1995), pp. 45–68.

57. James Fergusson (ed.), *Notes and Recollections of a Professional Life by the Late William Fergusson MD, Inspector General of Military Hospitals* (London: Longmans, Brown, Green and Longmans, 1846), p. 11.

58. Ibid., 12.

59. Ian Stuart Kelly, *Echoes of Success: Identity and the Highland Regiments* (Leiden: Brill, 2015), pp. 1–14.

60. See Murrary Pittock, *The Myth of the Jacobite Clans: The Jacobite Army in 1745* (Edinburgh: Edinburgh University Press, 2009).

61. Anon, ‘The Scottish Poor’, *The Morning Post*, 23 April 1859, p. 7.

62. Alexander Mackenzie, *The History of the Highland Clearances* (Glasgow: P.J. O’Callaghan, 1883), p. x.

63. Richard Tuthill Massey, *Analytical Ethnology* (London: Bailliere, 1855); G.K. Peatling, ‘Victorian Imperial Theorist? Goldwin Smith and Ireland’, in Peter Gray (ed), *Victoria’s Ireland? Irishness and Britishness, 1837–1901* (Dublin: Four Courts Press, 2004), pp. 27–36. – in anthropological review and journal 4 (1866).

64. Daniel Mackintosh, ‘Comparative Anthropology of England and Wales’ *Anthropological Review*, 4 (1866), p. 1–21, here 15; L. Perry Curtis, *Apes and Angels: The Irishman in Victorian Caricature* (Newton Abbot: David and Charles, 1971); Michael de Nie, *The Eternal Paddy: Irish Identity and the British Press, 1798–1882* (Madison: The University of Wisconsin Press, 2004).

65. Fergusson, *Notes and Recollections*, pp. 9–13.

66. Joseph Bernard Davis and John Thurnam, *Crania Britannica* (London: For Subscribers, 1865), p. 199.

67. See Miriam Rich, ‘The Curse of Civilised Woman: Race, Gender and the Pain of Childbirth in Nineteenth-Century American Medicine’, *Gender and History*, 28.1 (2016), pp. 57–76; Henry Connor, ‘The Use of Chloroform by British Army Surgeons during the Crimean War’, *Medical History*, 42 (1998), pp. 161–93; Niall Metcalfe, ‘The Influence of the Military on Civilian Uncertainty about Modern Anaesthesia Between its Origins in 1846 and the End of the Crimean War in 1856’, *Anaesthesia*, 60.6 (2005), pp. 594–601; John Shepherd, ‘“The Smart of the Knife” – Early Anaesthesia in the Services’, *JR Army Medical Corps*, 131 (1985), pp. 109–15.

68. Rawdon Macnamara, *Neligan’s Medicines* (Dublin: Fannin and Co., 1867), p. 187.

69. Ibid., 187–8.

70. Ibid., 502–3.

71. Thomas Watson, *Lecture on the Principles and Practice of Physic*, 2 vols (London: John Parker and Son, 1857), p. 1.120.

72. Fergusson, *Notes and Recollections*, p. 8.

73. John Mackinnon Robertson, *The Saxon and the Celt*, pp. 270–1.

74. Robert Knox, ‘Original Lectures on the Races of Men’, *Medical Times*, 19 (1848–9), pp. 17–20, 33–35, 49–50. See also Anon, ‘Ireland’ *The American Literary Magazine* 1 (August 1847), 77–93.

75. Anon, ‘Ireland’ *The American Literary Magazine*,1 (August 1847), pp. 77–93, here 86.

76. Ibid.

77. See for instance Mhamad Al-Hashimi, Simon Scott, Nicola Griffin-Teall and Jonathan Thompson, ‘Influence of Ethnicity on the Perception and Treatment of early Post-Operative Pain’, *British Journal of Pain*, 9.3 (2015), pp. 167–72.

78. Peter Mandler, ‘Race and “Nation”’.

## Permission

Both images are from Wellcome Images. Since I am funded by the Wellcome Trust, I believe that they will be available without permission in line with their policy.

